# Supplementary figures and images for: Polo-like kinase 4 (Plk4) potentiates anoikis-resistance of p53KO mammary epithelial cells by inducing a hybrid EMT phenotype
Source: Cell Death Dis. 2023 Feb 16;14(2):133. doi: 10.1038/s41419-023-05618-1 (PMC9935921; doi:10.1038/s41419-023-05618-1)

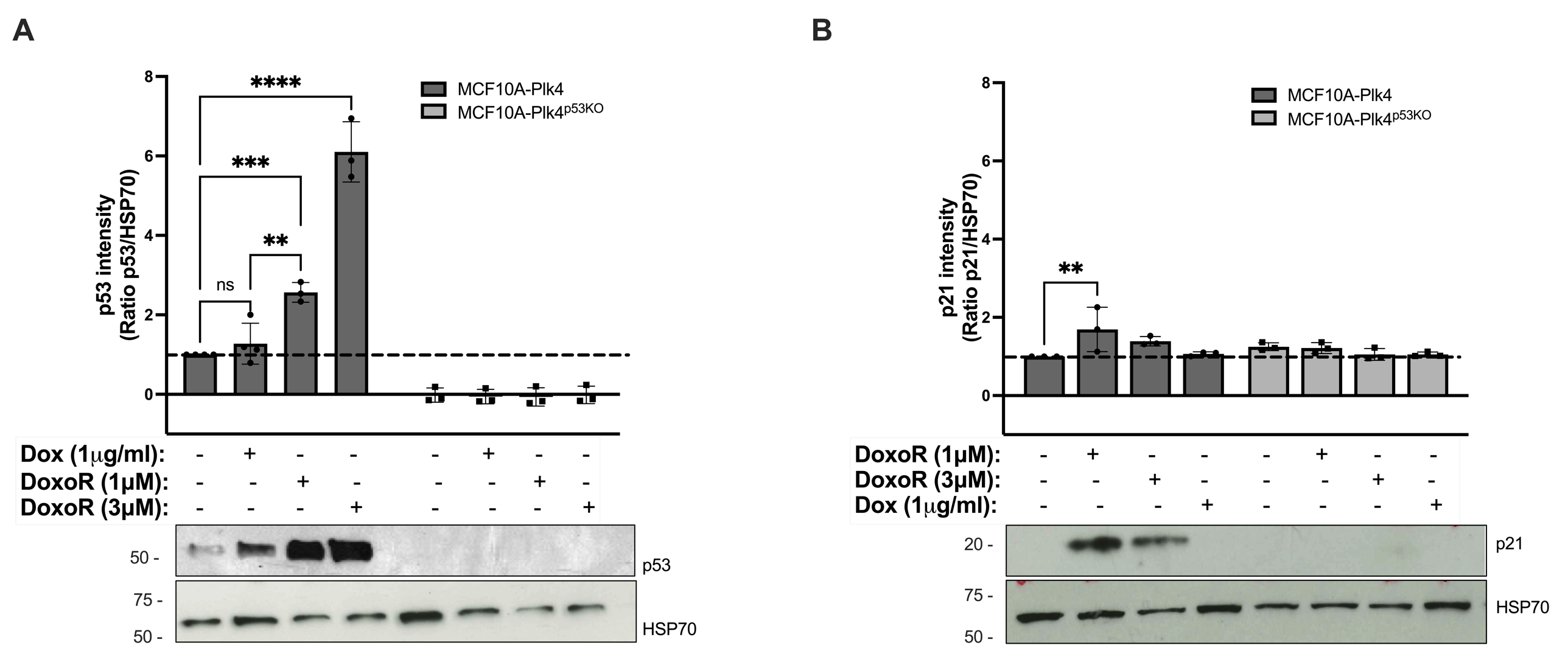

Supplement: Supplementary file 2 — Supplementary figure 1 [file 41419_2023_5618_MOESM2_ESM.tif]

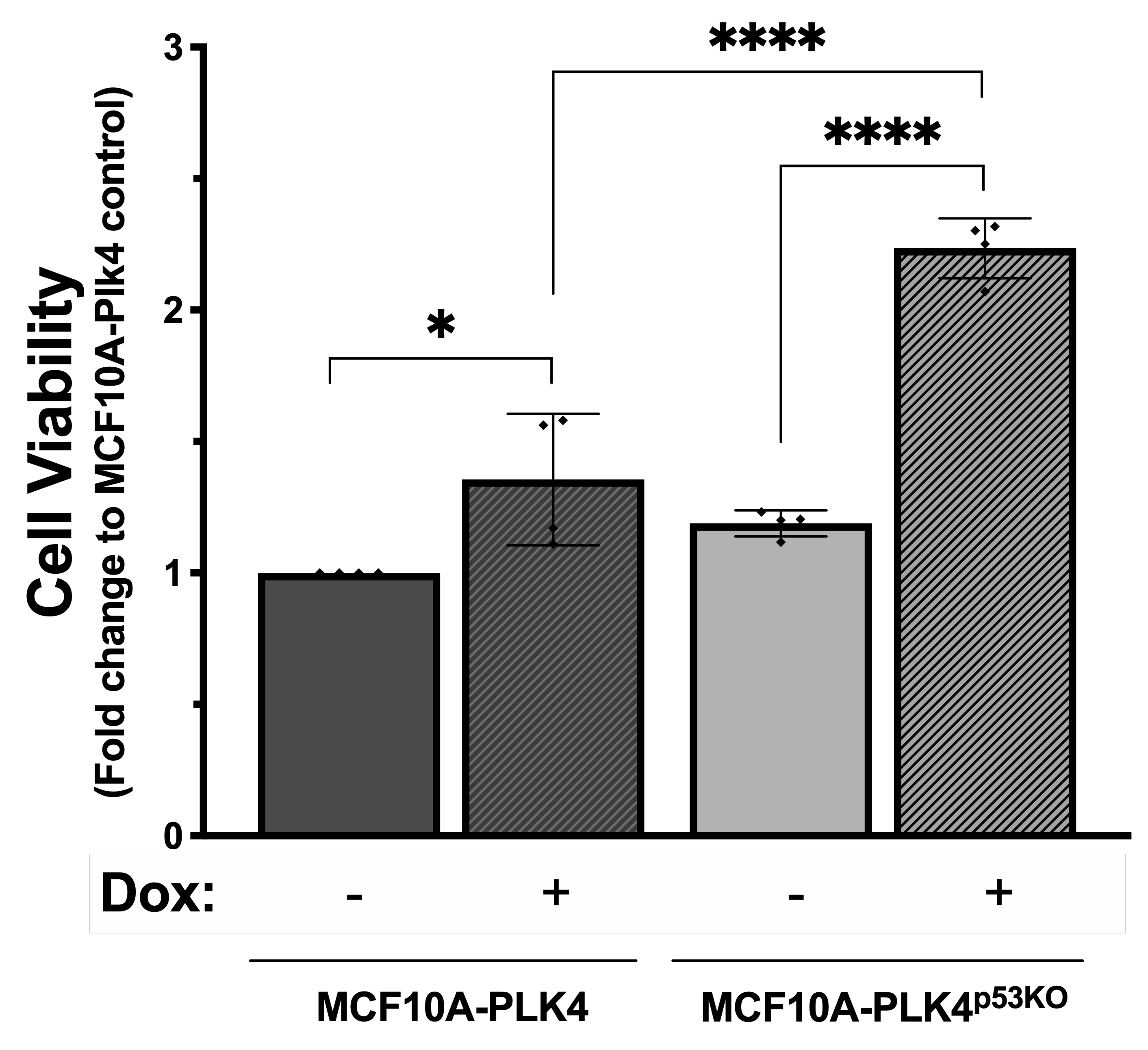

Supplement: Supplementary file 3 — Supplementary figure 2 [file 41419_2023_5618_MOESM3_ESM.tif]

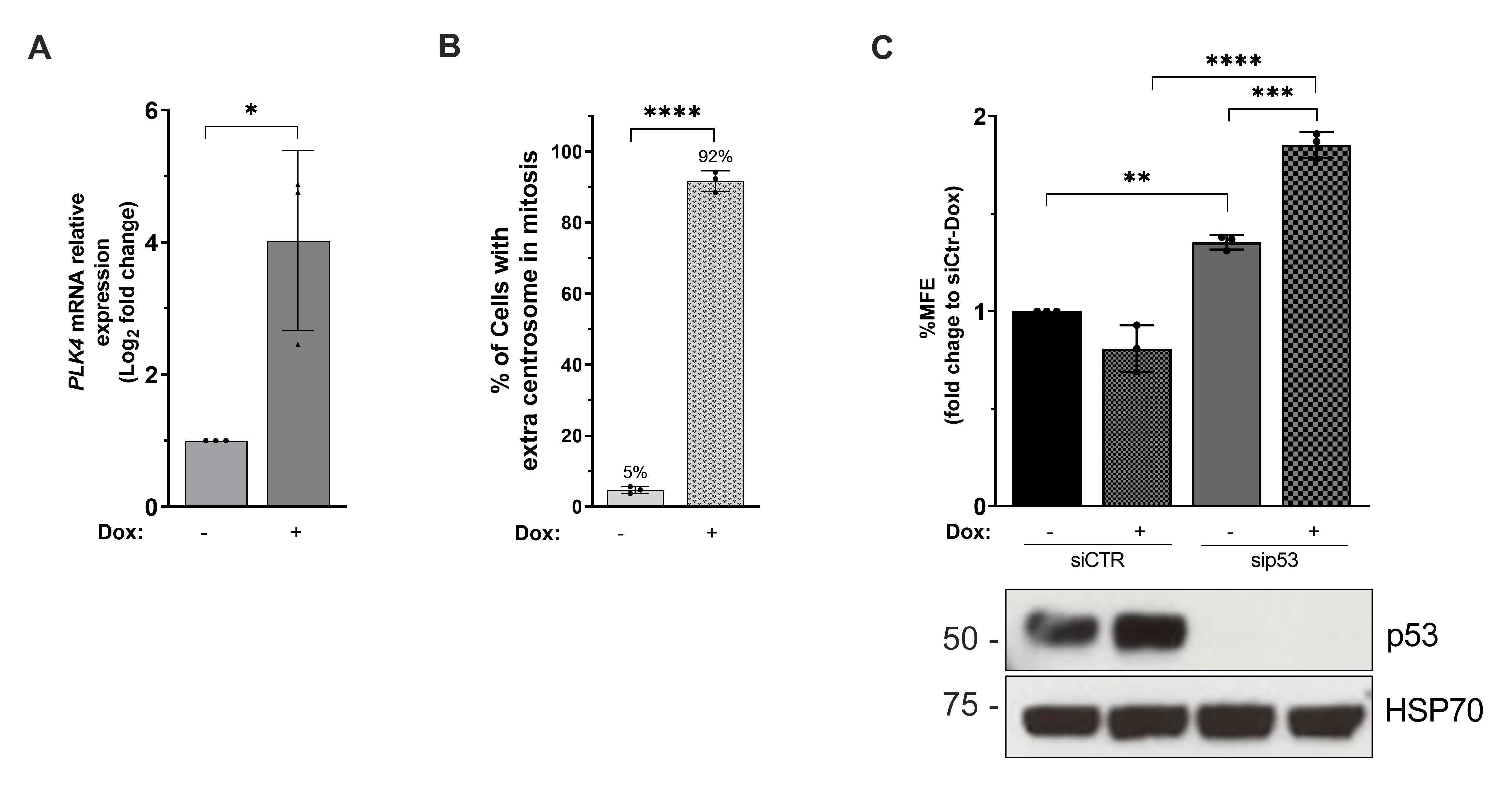

Supplement: Supplementary file 4 — Supplementary figure 3 [file 41419_2023_5618_MOESM4_ESM.tif]

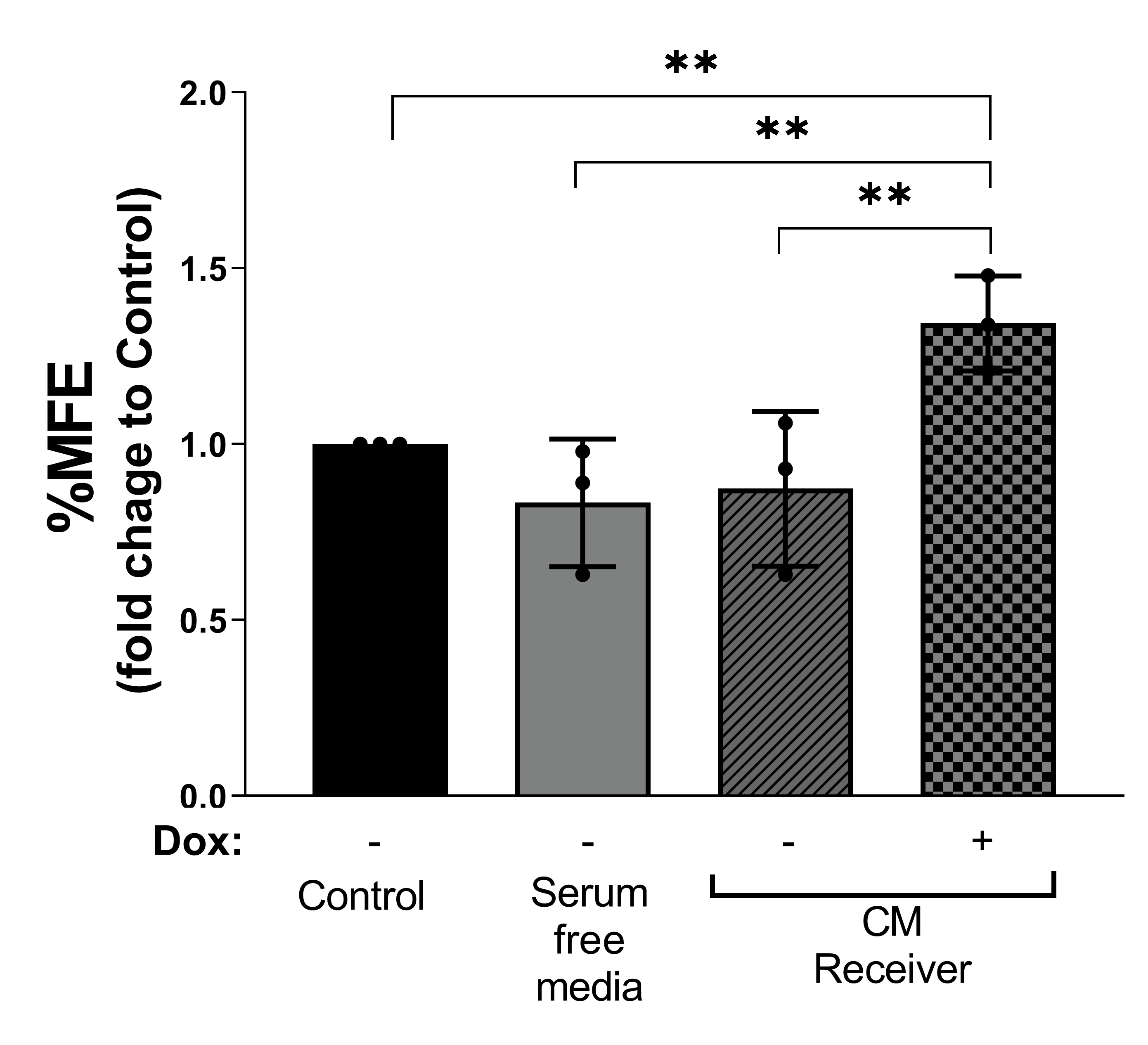

Supplement: Supplementary file 5 — Supplementary figure 4 [file 41419_2023_5618_MOESM5_ESM.tif]

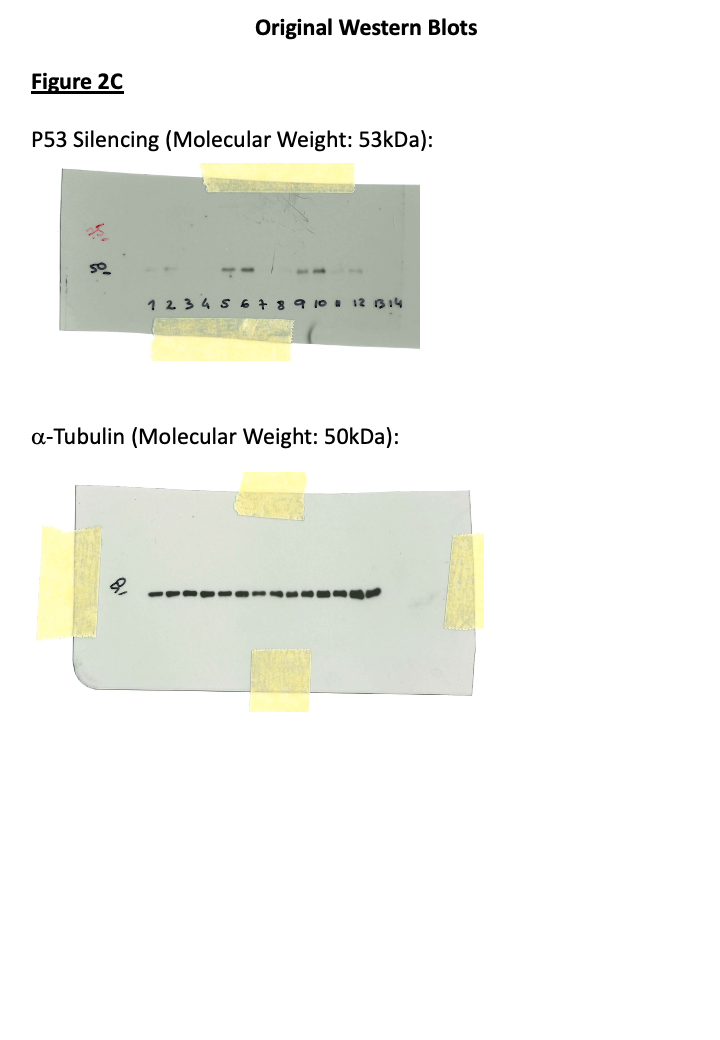

Supplement: Supplementary file 7 — Original Data File [file 41419_2023_5618_MOESM7_ESM.tif]
